# Crystal structure of [μ-1κ^2^ *C* ^1^,*C* ^4^:2(1,2,3,4-η)-1,2,3,4-tetra­phenyl­buta-1,3-diene-1,4-di­yl]bis­(tri­carbonyl­osmium)(*Os*—*Os*)

**DOI:** 10.1107/S2056989018011179

**Published:** 2018-08-14

**Authors:** Erin F. Rutledge, Kylie M. Wilson, Stephanie M. Martin, John W. Swartout, Ashley K. Archambeau, Emily R. Mikeska, Gregory L. Powell, Eric W. Reinheimer, Cynthia B. Powell

**Affiliations:** a Department of Chemistry & Biochemistry, Abilene Christian University, Abilene, TX 79699, USA; b Rigaku Oxford Diffraction, The Woodlands, Texas 77381, USA

**Keywords:** crystal structure, disorder, osmole complex, diene ligand, osmium carbon­yl, microwave heating

## Abstract

In the title complex C_34_H_20_O_6_Os_2_ or (μ-η^4^-C_4_Ph_4_)Os_2_(CO)_6_, one Os atom is part of a metalla­cyclo­penta­diene ring, while the second Os atom is π-bonded to the organic portion of this ring. The distance of 2.7494 (2) Å between the two Os atoms is typical of an Os—Os single bond. Three carbonyl ligands are attached to each Os atom and these six carbonyls adopt an eclipsed conformation. There are no bridging or semibridging CO groups. Two carbonyl ligands and all four phenyl groups are disordered over two slightly different positions for which each atom in the minor components is displaced less than 1 Å from the corresponding atom in the major components. The refined occupancies of the major com­ponents of the carbonyl ligands are 0.568 (16) and 0.625 (13), while those for the phenyl rings are 0.50 (3), 0.510 (12), 0.519 (18), and 0.568 (12).

## Abstract 2

A crystal structure of the osmole complex (μ-η^4^-C_4_Ph_4_)Os_2_(CO)_6_ revealed an eclipsed sawhorse mol­ecular geometry with no bridging or semi-bridging carbonyl ligands.

## Chemical context   

Metalla­cyclo­penta­diene complexes, known as metalloles, with the formula (μ-η^4^-C_4_ *R*_4_)*M*_2_(CO)_6_ are typically produced by C—C bond-coupling reactions of alkynes with group 8 metal carbonyls (Mathur *et al.*, 2014 ▸). These metalloles have been shown to adopt one of two possible geometries in the solid state, *i.e.* one in which the carbonyl ligands of the *M*_2_(CO)_6_ units are eclipsed in a so-called sawhorse conformation, or one in which the carbonyls are staggered with one CO semibridging the metal–metal bond. Ferroles (*M* = Fe) almost always adopt the staggered non-sawhorse conformation (Kumar *et al.*, 2014 ▸; Iyoda *et al.*, 1997 ▸; Jeannin *et al.*, 1994 ▸; Heim *et al.*, 1992 ▸; Daran & Jeannin, 1984 ▸), while ruthenoles (*M* = Ru) display an equal propensity to adopt either the sawhorse or the non-sawhorse conformation (Yang, 2014 ▸; Mathur *et al.*, 2008 ▸, 2014 ▸; Tunik *et al.*, 1997 ▸). Only two osmole (*M* = Os) complexes have been examined by X-ray crystallographic analysis, and both of them exhibit the sawhorse conformation. One of these is (μ-η^4^-2,3-di­methyl­butadiene)Os_2_(CO)_6_ (**I**) (see Scheme 1), which was prepared by reacting Os_3_(CO)_12_ with 2,3-di­methyl­butadiene, and in which the osma­cyclo­penta­diene ring contains H atoms in the 2,5-positions and methyl groups in the 3,4-positions (Dodge *et al.*, 1963 ▸). The other one is (μ-η^4^-FcC_2_-C≡CFc)_2_Os_2_(CO)_6_ (**II**, Fc is ferrocenyl), which was a product of the reaction of Os_3_(CO)_10_(NCMe)_2_ with 1,4-bis­(ferrocen­yl)butadiyne, and in which the osma­cyclo­penta­diene ring is substituted by ferrocen­yl–C≡C– groups in the 2,5-positions and by ferrocenyl groups in the 3,4-positions as a result of head-to-head coupling of the butadiyne starting material (Adams *et al.*, 2002 ▸).
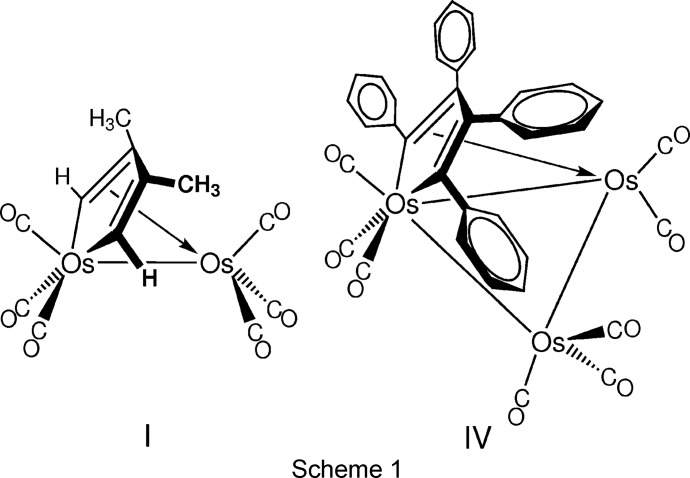


Our goal was to obtain the crystal structure of the title osmole (μ-η^4^-C_4_Ph_4_)Os_2_(CO)_6_ (**III**) (see Scheme 2) containing a tetra­phenyl­butadiene moiety, which was first reported over 46 years ago but which has never been structurally characterized (Gambino *et al.*, 1971 ▸). Gambino *et al.* prepared **III** by a three-step process: Os_3_(CO)_12_ was heated with di­phenyl­acetyl­ene (tolan) to produce Os_3_(CO)_8_(C_4_Ph_4_) (**IV**), which was treated with CO to yield Os_3_(CO)_9_(C_4_Ph_4_) (**V**). This was then treated with excess CO to produce **III**. The overall yield for **III** based on Os_3_(CO)_12_ was not mentioned, but it was clearly less than 4% since the yields for the first two steps were reported to be about 10 and 40%, respectively. In order to obtain a significant qu­antity of **III** for crystal growing attempts, we sought a higher yield method of preparing this osmole complex. We turned to microwave heating since it had been shown to offer improved efficiency for the preparation of certain other osmium carbonyl complexes (Johnson & Powell, 2008 ▸; Leadbeater & Shoemaker, 2008 ▸; Jung *et al.*, 2012 ▸; Pyper *et al.*, 2013 ▸).
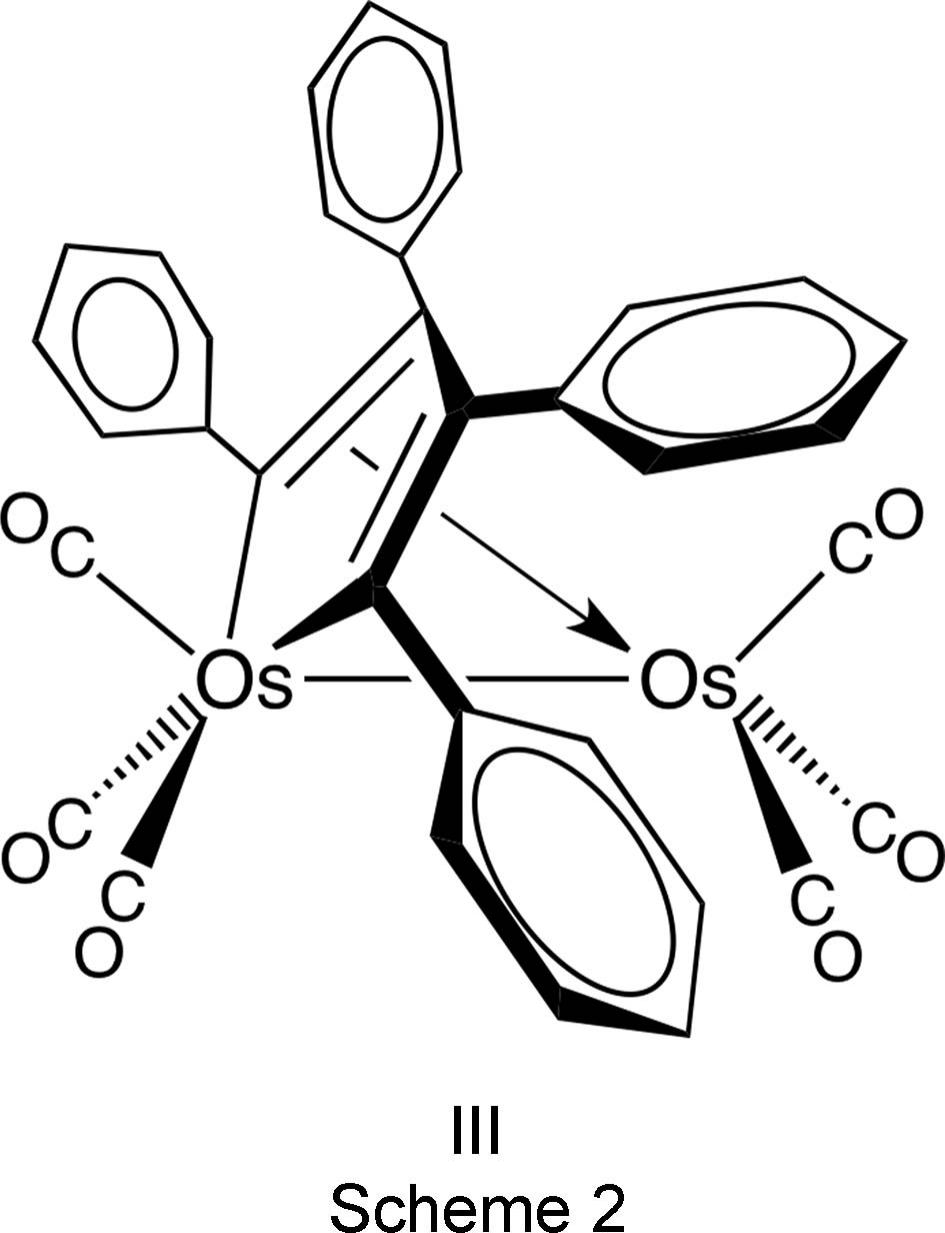


## Structural commentary   

**Figure 1:**
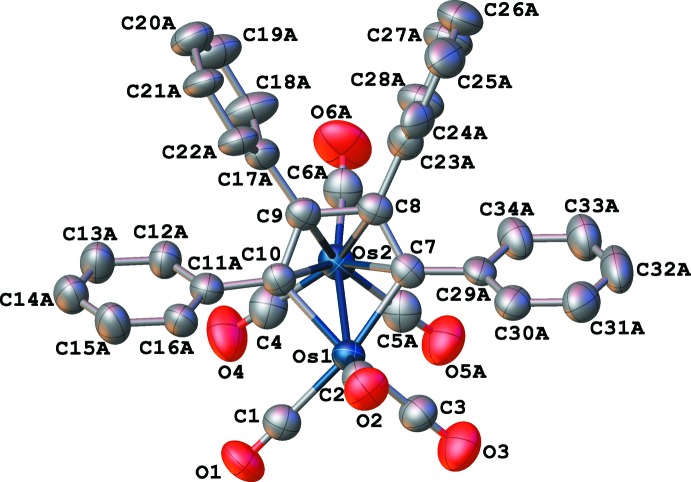
The mol­ecular structure of the title compound, showing the positions of the major phenyl-ring and carbonyl-ligand components, as well as the atom-labelling scheme. Displacement ellipsoids are shown at the 50% probability level and H atoms have been omitted for clarity.

**Figure 2:**
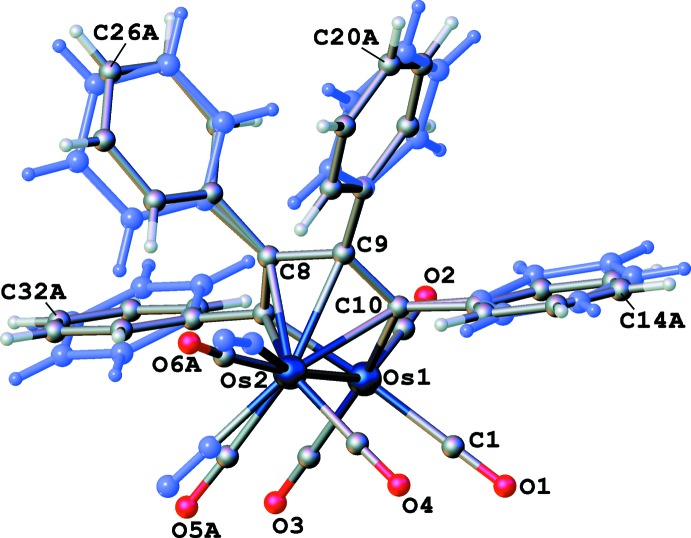
A ball-and-stick view of the asymmetric unit of **III**, with partial atom labeling. All components of the disordered carbonyl ligands and phenyl rings are shown (the minor ones in pale blue).

The mol­ecular structure of compound **III** is illustrated in Fig. 1 ▸. All four phenyl rings are disordered over two slightly different orientations (Fig. 2 ▸), and the refined occupancies of the major components are 0.50 (3), 0.510 (12), 0.519 (18), and 0.568 (12). Two of the carbonyl ligands are also disordered over two slightly different positions and the occupancies of the major components are 0.568 (16) and 0.625 (13). Each C or O atom in the minor components is displaced less than 1 Å from its counterpart in the major components. The geometrical features of the central portion of **III** are quite similar to those of the two (μ-η^4^-C_4_ *R*_4_)Os_2_(CO)_6_ osmoles that have been previously characterized by X-ray crystallography, with planar osma­cyclo­penta­diene rings and eclipsed sawhorse conformations of the carbonyls. Thus, there are no bridging or semibridging CO ligands. The *R* groups (phenyl rings) in **III** are inter­mediate in size compared to those of the other two osmoles, one of which (*i.e.* **I**) had small butadiene substituents of H and Me, while the other (*i.e.* **II**) had large substituents of Fc—C≡C— and Fc. The Os—Os bond lengths of 2.74 Å for **I**, 2.7494 (2) Å for **III**, and 2.7556 (7) Å for **II** might reflect an inverse correlation between the strength of the metal–metal bond and the steric bulk of the butadiene substituents, although the rudimentary nature of the crystal structure report for **I** precludes a definitive conclusion concerning this trend (the only bond length included in the description of the structure of **I** was the Os—Os distance and no s.u. value was given). The average bond lengths between the Os atoms that lie within the metallacyclpenta­diene rings and the 2,5-C atoms of the rings are 2.09 (1) Å for **II** and 2.10 (1) Å for **III**, while the other Os atoms in **II** and **III** have an average distance of 2.31 (4) and 2.32 (4) Å, respectively, from the four C atoms in the metallacyclpenta­diene rings. The central C—C distances in the C_4_ *R*_4_ groups are 1.48 (1) Å for **II** and 1.461 (5) Å for **III**, and these are both longer than the other two C—C distances on either side of them [average of 1.42 (1) Å for **II** and 1.420 (5) Å for **III**], supporting the designation of these groups as dienes. There are five unique torsion angles within each metallacyclpenta­diene ring, and the average values of these are 8° for **II** and 0.7° for **III**. Thus, the planarity of the metallacyclpenta­diene ring in **III** is less distorted than it is in **II**, which is most likely a consequence of the smaller steric bulk of the *R* groups in **III**.

## Database survey   

A search of the Cambridge Structural Database (Version 5.39, last update February 2018; Groom *et al.*, 2016 ▸) for metallole complexes of the type (μ-η^4^-C_4_ *R*_4_)*M*_2_(CO)_6_, where *M* is any transition metal, gave 14 hits. The only hit containing Os atoms was complex **II** with a sawhorse conformation and no bridging carbonyl ligands. Eight of the hits were for ruthenoles, four with non-sawhorse conformations and semibridging CO ligands and four with sawhorse conformations without bridging carbonyls. The five remaining hits were for ferroles, all of which have semibridging CO ligands.

## Supra­molecular features   

**Figure 3:**
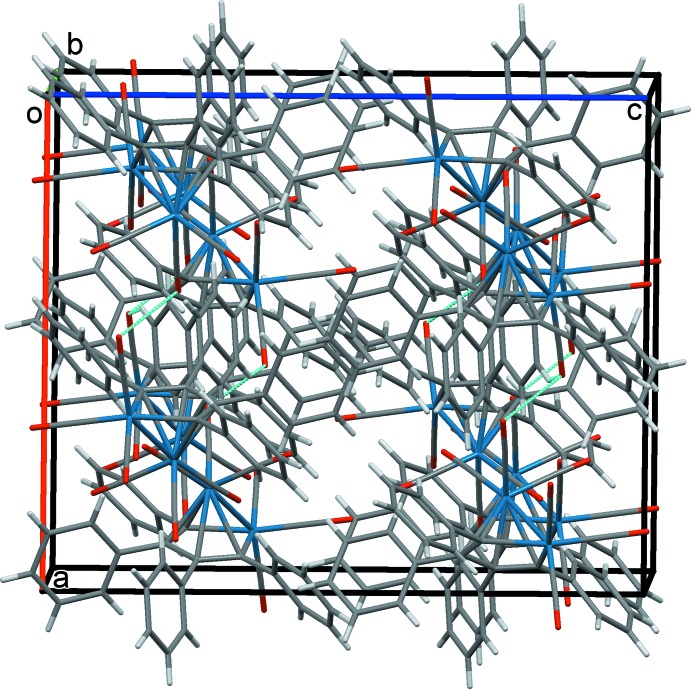
The overall packing of **III**, viewed along the *b*-axis direction.

There are only two inter­molecular nonbonding distances in the structure of **III** that are shorter than the sum of the van der Waals radii. A weak C19*A*—H19*A*⋯O2^i^ hydrogen bond (Table 1 ▸) and a close O2⋯O5^ii^ contact of 2.941 (9) Å [symmetry code: (ii) −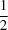 + *x*, 1 − *y*, z]. These combine to stack mol­ecules of **III** along the direction of the *b* axis of the unit cell (Fig. 3 ▸).

## Synthesis and crystallization   

Dodeca­carbonyl­triosmium(0) (100 mg, 0.110 mmol) and MeCN (8 ml) were placed in a 35 ml glass reaction vessel, then sealed with a PTFE cap and placed in a CEM Discover-SP microwave reactor. The mixture was stirred and heated at 403 K for 8 min to yield a green solution in which the major component was known to be Os_3_(CO)_11_(NCMe), as noted in a previous report (Jung *et al.*, 2009 ▸). The reaction vessel was removed from the microwave reactor and allowed to cool to room temperature. Di­phenyl­acetyl­ene (118 mg, 0.662 mmol) was added to the vessel and it was returned to the microwave reactor. This solution was stirred and heated at 433 K for 6 min. The solvent was removed by rotary evaporation, then the residue was dissolved in CH_2_Cl_2_ and subjected to thin-layer chromatography (TLC) using an eluent of 1:1 (*v*/*v*) hexa­nes/CH_2_Cl_2_. Three yellow bands were collected. The top band consisted of 34.1 mg (22.8% yield) of complex **III**. IR (ν_CO_, hexa­ne): 2081 (*s*), 2051 (*vs*), 2018 (*m*), 1998 (*s*), and 1968 (*m*) cm^−1^. The second band consisted of a mixture of complex **III** and an unidentified product. The third band consisted of 4.1 mg (3.2% yield) of complex **IV**. Crystals of **III** were grown by slow evaporation of an *n*-hexane solution at room temperature.

## Refinement   

Crystal data, data collection and structure refinement details are summarized in Table 2 ▸. The C atoms in the four phenyl rings were disordered over two slightly different orientations. Each phenyl ring was split into two components (*A* and *B*), which were refined as rigid hexa­gons. H atoms were included in idealized positions and allowed to ride on their parent atoms: C—H = 0.95 Å with *U*_iso_(H) = 1.2*U*_eq_(C). The refined occupancy ratios were C11–C16 0.519 (18):0.481 (18), C17–C22 0.50 (3):0.50 (3), C23–C28 0.568 (12):0.432 (12), and C29–C34 0.510 (12):0.490 (12). Two of the CO ligands were also disordered over two slightly different positions. The refined occupancy ratios for these were C5≡O5 0.625 (13):0.375 (13) and C6≡O6 0.568 (16):0.432 (16). The best data were obtained at room temperature. X-ray data were collected on the same crystal and several other crystals of **III** at lower temperatures, but as the temperature decreased, the disorder of the phenyl rings and carbonyl ligands became more extensive and increasingly difficult to model.

## Supplementary Material

Crystal structure: contains datablock(s) I. DOI: 10.1107/S2056989018011179/sj5562sup1.cif

Structure factors: contains datablock(s) I. DOI: 10.1107/S2056989018011179/sj5562Isup2.hkl

Supporting information file. DOI: 10.1107/S2056989018011179/sj5562Isup3.mol

CCDC reference: 1847589

Additional supporting information:  crystallographic information; 3D view; checkCIF report

## Figures and Tables

**Table 1:** Hydrogen-bond geometry (Å, °)

*D*—H⋯*A*	*D*—H	H⋯*A*	*D*⋯*A*	*D*—H⋯*A*
C19*A*—H19*A*⋯O2^i^	0.93	2.60	3.363 (12)	139

Symmetry code: (i) 
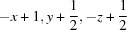
.

**Table 2:** Experimental details

Crystal data	
Chemical formula	[Os_2_(C_28_H_20_)(CO)_6_]
*M*_r_	904.90
Crystal system, space group	Monoclinic, *I*2/*a*
Temperature (K)	298
*a*, *b*, *c* (Å)	15.3471 (1), 21.2919 (2), 18.5565 (1)
β (°)	90.298 (1)
*V* (Å^3^)	6063.60 (8)
*Z*	8
Radiation type	Cu *K*α
μ (mm^−1^)	15.95
Crystal size (mm)	0.15 × 0.08 × 0.07

Data collection	
Diffractometer	Rigaku OD SuperNova Dual source diffractometer with an AtlasS2 detector
Absorption correction	Gaussian (*CrysAlis PRO*; Rigaku OD, 2018 ▸)
*T*_min_, *T*_max_	0.296, 0.506
No. of measured, independent and observed [*I* > 2σ(*I*)] reflections	27474, 5352, 5118
*R*_int_	0.021
(sin θ/λ)_max_ (Å^−1^)	0.595

Refinement	
*R*[*F* ^2^ > 2σ(*F* ^2^)], *wR*(*F* ^2^), *S*	0.019, 0.046, 1.05
No. of reflections	5352
No. of parameters	512
No. of restraints	167
H-atom treatment	H-atom parameters constrained
Δρ_max_, Δρ_min_ (e Å^−3^)	1.18, −0.94

Computer programs: *CrysAlis PRO* (Rigaku OD, 2018 ▸), *SHELXT* (Sheldrick, 2015*a* ▸), *SHELXL2018* (Sheldrick, 2015*b* ▸), *OLEX2* (Dolomanov *et al.*, 2009 ▸) and *Mercury* (Macrae *et al.*, 2008 ▸).

## supplementary crystallographic information

### Crystal data

**Table:**

[Os_2_(C_28_H_20_)(CO)_6_]	*F*(000) = 3392
*M**_r_* = 904.90	*D*_x_ = 1.982 Mg m^−^^3^
Monoclinic, *I*2/*a*	Cu *K*α radiation, λ = 1.54184 Å
*a* = 15.3471 (1) Å	Cell parameters from 20186 reflections
*b* = 21.2919 (2) Å	θ = 4.7–73.5°
*c* = 18.5565 (1) Å	µ = 15.95 mm^−^^1^
β = 90.298 (1)°	*T* = 298 K
*V* = 6063.60 (8) Å^3^	Block, red
*Z* = 8	0.14 × 0.08 × 0.07 mm

### Data collection

**Table:**

Rigaku OD SuperNova Dual source diffractometer with an AtlasS2 detector	5118 reflections with *I* > 2σ(*I*)
Detector resolution: 5.2387 pixels mm^-1^	*R*_int_ = 0.021
ω scans	θ_max_ = 66.6°, θ_min_ = 4.8°
Absorption correction: gaussian (CrysAlis PRO; Rigaku OD, 2018)	*h* = −18→18
*T*_min_ = 0.296, *T*_max_ = 0.506	*k* = −24→25
27474 measured reflections	*l* = −22→22
5352 independent reflections	

### Refinement

**Table:**

Refinement on *F*^2^	Hydrogen site location: inferred from neighbouring sites
Least-squares matrix: full	H-atom parameters constrained
*R*[*F*^2^ > 2σ(*F*^2^)] = 0.019	*w* = 1/[σ^2^(*F*_o_^2^) + (0.0207*P*)^2^ + 11.4338*P*] where *P* = (*F*_o_^2^ + 2*F*_c_^2^)/3
*wR*(*F*^2^) = 0.046	(Δ/σ)_max_ = 0.002
*S* = 1.05	Δρ_max_ = 1.18 e Å^−^^3^
5352 reflections	Δρ_min_ = −0.93 e Å^−^^3^
512 parameters	Extinction correction: SHELXL2018 (Sheldrick, 2015b), Fc^*^=kFc[1+0.001xFc^2^λ^3^/sin(2θ)]^-1/4^
167 restraints	Extinction coefficient: 0.000017 (2)

### Special details

**Table:**

Geometry. All esds (except the esd in the dihedral angle between two l.s. planes) are estimated using the full covariance matrix. The cell esds are taken into account individually in the estimation of esds in distances, angles and torsion angles; correlations between esds in cell parameters are only used when they are defined by crystal symmetry. An approximate (isotropic) treatment of cell esds is used for estimating esds involving l.s. planes.

### Fractional atomic coordinates and isotropic or equivalent isotropic displacement parameters (Å^2^)

**Table:**

	*x*	*y*	*z*	*U*_iso_*/*U*_eq_	Occ. (<1)
Os1	0.62389 (2)	0.49607 (2)	0.15025 (2)	0.04580 (5)	
Os2	0.71430 (2)	0.58750 (2)	0.22382 (2)	0.05374 (6)	
O2	0.46073 (18)	0.41761 (13)	0.13217 (17)	0.0730 (7)	
O1	0.6492 (2)	0.52162 (16)	−0.01161 (15)	0.0834 (8)	
C10	0.5756 (2)	0.58624 (14)	0.16889 (17)	0.0475 (7)	
O3	0.7493 (2)	0.38377 (17)	0.1469 (2)	0.0934 (9)	
C7	0.6298 (2)	0.50278 (15)	0.26329 (17)	0.0480 (7)	
O4	0.7858 (2)	0.64993 (18)	0.08904 (19)	0.1020 (11)	
C9	0.5694 (2)	0.60595 (15)	0.24180 (17)	0.0495 (7)	
C8	0.5995 (2)	0.55959 (15)	0.29438 (17)	0.0488 (7)	
C1	0.6400 (2)	0.51209 (17)	0.0480 (2)	0.0587 (8)	
C2	0.5222 (2)	0.44711 (16)	0.13780 (18)	0.0529 (8)	
C3	0.7024 (3)	0.42495 (19)	0.1475 (2)	0.0630 (9)	
O5A	0.8706 (4)	0.4947 (4)	0.2289 (5)	0.0934 (9)	0.625 (13)
C4	0.7605 (3)	0.6268 (2)	0.1397 (2)	0.0697 (10)	
C22B	0.4324 (10)	0.6596 (6)	0.2462 (12)	0.0648 (18)	0.50 (3)
H22B	0.412221	0.626176	0.218497	0.078*	0.50 (3)
C21B	0.3749 (8)	0.7060 (8)	0.2687 (11)	0.078 (4)	0.50 (3)
H21B	0.316295	0.703623	0.256037	0.094*	0.50 (3)
C20B	0.4050 (11)	0.7559 (7)	0.3101 (9)	0.081 (4)	0.50 (3)
H20B	0.366592	0.786977	0.325197	0.098*	0.50 (3)
C19B	0.4927 (12)	0.7595 (6)	0.3291 (9)	0.088 (3)	0.50 (3)
H19B	0.512814	0.792885	0.356818	0.106*	0.50 (3)
C18B	0.5501 (9)	0.7131 (6)	0.3066 (11)	0.072 (3)	0.50 (3)
H18B	0.608741	0.715439	0.319278	0.086*	0.50 (3)
C17B	0.5200 (9)	0.6631 (6)	0.2652 (13)	0.0575 (8)	0.50 (3)
C16B	0.4631 (13)	0.6056 (7)	0.0780 (10)	0.072 (5)	0.481 (18)
H16B	0.443788	0.565370	0.089217	0.087*	0.481 (18)
C15B	0.4183 (10)	0.6415 (7)	0.0274 (8)	0.072 (3)	0.481 (18)
H15B	0.368909	0.625307	0.004835	0.086*	0.481 (18)
C14B	0.4472 (10)	0.7016 (7)	0.0106 (7)	0.072 (2)	0.481 (18)
H14B	0.417198	0.725577	−0.023226	0.086*	0.481 (18)
C13B	0.5210 (10)	0.7257 (6)	0.0443 (8)	0.075 (2)	0.481 (18)
H13B	0.540366	0.765911	0.033095	0.090*	0.481 (18)
C12B	0.5659 (12)	0.6898 (8)	0.0949 (10)	0.071 (3)	0.481 (18)
H12B	0.615245	0.705975	0.117478	0.086*	0.481 (18)
C11B	0.5369 (14)	0.6297 (8)	0.1117 (11)	0.0547 (11)	0.481 (18)
C5A	0.8115 (4)	0.5307 (3)	0.2256 (5)	0.0632 (9)	0.625 (13)
O6A	0.8001 (12)	0.6787 (5)	0.3297 (7)	0.114 (4)	0.568 (16)
C6A	0.7709 (6)	0.6429 (5)	0.2911 (4)	0.0632 (9)	0.568 (16)
C34B	0.7313 (6)	0.4375 (5)	0.3494 (7)	0.075 (4)	0.490 (12)
H34B	0.773766	0.468676	0.347749	0.091*	0.490 (12)
C33B	0.7459 (6)	0.3833 (6)	0.3894 (7)	0.081 (3)	0.490 (12)
H33B	0.797998	0.378124	0.414442	0.098*	0.490 (12)
C32B	0.6824 (8)	0.3367 (5)	0.3919 (7)	0.085 (4)	0.490 (12)
H32B	0.692144	0.300440	0.418622	0.102*	0.490 (12)
C31B	0.6045 (8)	0.3444 (6)	0.3544 (8)	0.073 (3)	0.490 (12)
H31B	0.562058	0.313306	0.356109	0.088*	0.490 (12)
C30B	0.5900 (6)	0.3987 (7)	0.3145 (8)	0.060 (2)	0.490 (12)
H30B	0.537825	0.403857	0.289416	0.072*	0.490 (12)
C29B	0.6534 (7)	0.4452 (6)	0.3120 (7)	0.058 (4)	0.490 (12)
C6B	0.7491 (8)	0.6609 (5)	0.2770 (6)	0.0637 (10)	0.432 (16)
O6B	0.7851 (18)	0.7002 (7)	0.3059 (10)	0.129 (7)	0.432 (16)
C12A	0.5787 (11)	0.6907 (7)	0.1098 (9)	0.063 (3)	0.519 (18)
H12A	0.624330	0.701954	0.140324	0.076*	0.519 (18)
C13A	0.5446 (9)	0.7342 (6)	0.0615 (7)	0.075 (2)	0.519 (18)
H13A	0.567412	0.774662	0.059736	0.090*	0.519 (18)
C14A	0.4763 (9)	0.7174 (6)	0.0160 (6)	0.072 (2)	0.519 (18)
H14A	0.453502	0.746534	−0.016346	0.086*	0.519 (18)
C15A	0.4422 (10)	0.6570 (6)	0.0187 (7)	0.075 (3)	0.519 (18)
H15A	0.396510	0.645698	−0.011840	0.090*	0.519 (18)
C16A	0.4763 (12)	0.6134 (6)	0.0669 (9)	0.060 (2)	0.519 (18)
H16A	0.453427	0.572989	0.068748	0.072*	0.519 (18)
C11A	0.5445 (13)	0.6303 (7)	0.1125 (10)	0.0547 (11)	0.519 (18)
C18A	0.5678 (8)	0.7125 (6)	0.2967 (13)	0.092 (6)	0.50 (3)
H18A	0.628236	0.710186	0.300154	0.111*	0.50 (3)
C19A	0.5243 (12)	0.7650 (5)	0.3228 (10)	0.088 (3)	0.50 (3)
H19A	0.555515	0.797745	0.343661	0.106*	0.50 (3)
C20A	0.4340 (12)	0.7685 (5)	0.3176 (9)	0.074 (4)	0.50 (3)
H20A	0.404865	0.803555	0.335076	0.089*	0.50 (3)
C21A	0.3873 (8)	0.7195 (7)	0.2864 (10)	0.069 (3)	0.50 (3)
H21A	0.326936	0.721807	0.282985	0.083*	0.50 (3)
C22A	0.4309 (9)	0.6670 (6)	0.2604 (11)	0.0647 (18)	0.50 (3)
H22A	0.399656	0.634247	0.239478	0.078*	0.50 (3)
C17A	0.5212 (9)	0.6635 (6)	0.2655 (13)	0.0574 (8)	0.50 (3)
C23A	0.5812 (6)	0.5722 (4)	0.37263 (15)	0.061 (5)	0.568 (12)
C24A	0.5003 (6)	0.5547 (5)	0.3992 (3)	0.059 (3)	0.568 (12)
H24A	0.459786	0.535424	0.368955	0.071*	0.568 (12)
C25A	0.4798 (6)	0.5660 (5)	0.4709 (4)	0.084 (4)	0.568 (12)
H25A	0.425605	0.554296	0.488652	0.101*	0.568 (12)
C26A	0.5403 (7)	0.5948 (5)	0.51606 (19)	0.096 (2)	0.568 (12)
H26A	0.526582	0.602338	0.564040	0.115*	0.568 (12)
C27A	0.6213 (6)	0.6123 (5)	0.4895 (3)	0.090 (3)	0.568 (12)
H27A	0.661742	0.631508	0.519729	0.108*	0.568 (12)
C28A	0.6417 (5)	0.6009 (5)	0.4178 (3)	0.078 (3)	0.568 (12)
H28A	0.695926	0.612637	0.400032	0.093*	0.568 (12)
C23B	0.5893 (7)	0.5673 (4)	0.37446 (15)	0.051 (5)	0.432 (12)
C24B	0.5044 (6)	0.5753 (6)	0.3986 (4)	0.052 (3)	0.432 (12)
H24B	0.458286	0.574581	0.365926	0.062*	0.432 (12)
C25B	0.4886 (7)	0.5844 (6)	0.4715 (5)	0.069 (4)	0.432 (12)
H25B	0.431784	0.589804	0.487654	0.083*	0.432 (12)
C26B	0.5576 (9)	0.5856 (6)	0.5203 (2)	0.096 (2)	0.432 (12)
H26B	0.546937	0.591666	0.569120	0.115*	0.432 (12)
C27B	0.6424 (8)	0.5776 (7)	0.4962 (3)	0.090 (3)	0.432 (12)
H27B	0.688594	0.578303	0.528860	0.108*	0.432 (12)
C28B	0.6583 (6)	0.5684 (6)	0.4233 (4)	0.070 (3)	0.432 (12)
H28B	0.715099	0.563081	0.407133	0.084*	0.432 (12)
C30A	0.6142 (7)	0.3945 (7)	0.3058 (6)	0.059 (2)	0.510 (12)
H30A	0.571537	0.388665	0.270733	0.071*	0.510 (12)
C31A	0.6356 (9)	0.3459 (5)	0.3526 (7)	0.073 (3)	0.510 (12)
H31A	0.607114	0.307437	0.348851	0.088*	0.510 (12)
C32A	0.6994 (8)	0.3547 (5)	0.4051 (6)	0.090 (4)	0.510 (12)
H32A	0.713633	0.322097	0.436414	0.108*	0.510 (12)
C33A	0.7419 (6)	0.4121 (6)	0.4108 (5)	0.081 (3)	0.510 (12)
H33A	0.784575	0.417984	0.445859	0.098*	0.510 (12)
C34A	0.7206 (7)	0.4608 (5)	0.3640 (6)	0.069 (3)	0.510 (12)
H34A	0.748998	0.499213	0.367743	0.083*	0.510 (12)
C29A	0.6567 (7)	0.4520 (5)	0.3115 (6)	0.048 (3)	0.510 (12)
C5B	0.8208 (5)	0.5440 (6)	0.2457 (8)	0.0632 (9)	0.375 (13)
O5B	0.8734 (7)	0.5156 (7)	0.2566 (9)	0.0934 (9)	0.375 (13)

### Atomic displacement parameters (Å^2^)

**Table:**

	*U*^11^	*U*^22^	*U*^33^	*U*^12^	*U*^13^	*U*^23^
Os1	0.04688 (9)	0.04677 (9)	0.04374 (8)	−0.00282 (5)	0.00123 (6)	−0.00239 (5)
Os2	0.04855 (9)	0.05941 (10)	0.05324 (9)	−0.01425 (6)	−0.00124 (6)	−0.00057 (6)
O2	0.0593 (16)	0.0678 (17)	0.092 (2)	−0.0166 (13)	−0.0031 (14)	−0.0063 (14)
O1	0.098 (2)	0.104 (2)	0.0482 (15)	−0.0096 (18)	0.0066 (14)	0.0067 (14)
C10	0.0485 (17)	0.0462 (17)	0.0476 (16)	−0.0076 (13)	−0.0044 (13)	−0.0021 (13)
O3	0.0727 (14)	0.090 (2)	0.117 (2)	0.0196 (13)	0.0055 (15)	0.0113 (16)
C7	0.0424 (16)	0.0548 (18)	0.0468 (17)	−0.0099 (13)	0.0012 (13)	0.0021 (13)
O4	0.089 (2)	0.119 (3)	0.098 (2)	0.003 (2)	0.0261 (19)	0.048 (2)
C9	0.0509 (17)	0.0466 (17)	0.0511 (17)	−0.0093 (14)	−0.0021 (14)	−0.0054 (13)
C8	0.0465 (16)	0.0533 (18)	0.0465 (16)	−0.0076 (14)	0.0010 (13)	−0.0040 (14)
C1	0.057 (2)	0.060 (2)	0.058 (2)	−0.0059 (16)	0.0007 (16)	−0.0029 (16)
C2	0.056 (2)	0.0510 (18)	0.0516 (18)	0.0026 (16)	0.0015 (15)	−0.0018 (14)
C3	0.058 (2)	0.065 (2)	0.066 (2)	0.0062 (18)	0.0038 (17)	−0.0021 (17)
O5A	0.0727 (14)	0.090 (2)	0.117 (2)	0.0196 (13)	0.0055 (15)	0.0113 (16)
C4	0.056 (2)	0.069 (2)	0.084 (3)	−0.0039 (18)	0.0023 (19)	0.010 (2)
C22B	0.079 (2)	0.053 (3)	0.063 (6)	0.010 (2)	0.005 (3)	−0.019 (2)
C21B	0.100 (5)	0.068 (6)	0.067 (9)	0.026 (5)	−0.002 (5)	−0.029 (5)
C20B	0.121 (10)	0.053 (6)	0.070 (7)	0.023 (6)	−0.005 (8)	−0.021 (5)
C19B	0.103 (9)	0.059 (3)	0.102 (5)	−0.009 (5)	0.024 (7)	−0.028 (3)
C18B	0.095 (6)	0.057 (6)	0.062 (5)	−0.008 (5)	0.006 (6)	−0.016 (4)
C17B	0.0798 (19)	0.0450 (18)	0.0477 (17)	−0.0004 (16)	0.0040 (16)	−0.0042 (14)
C16B	0.083 (7)	0.068 (5)	0.066 (8)	−0.003 (4)	−0.016 (6)	0.009 (5)
C15B	0.086 (7)	0.065 (5)	0.064 (6)	0.003 (4)	−0.014 (5)	0.005 (4)
C14B	0.095 (7)	0.062 (5)	0.059 (3)	0.003 (4)	−0.015 (4)	0.002 (4)
C13B	0.100 (7)	0.057 (4)	0.068 (6)	0.000 (3)	−0.019 (4)	0.006 (4)
C12B	0.085 (7)	0.062 (3)	0.067 (7)	−0.005 (4)	−0.013 (5)	0.011 (4)
C11B	0.065 (3)	0.0536 (18)	0.0453 (17)	0.0068 (19)	0.0034 (18)	−0.0023 (13)
C5A	0.0470 (15)	0.0682 (19)	0.074 (3)	−0.0123 (11)	−0.0116 (13)	0.0018 (15)
O6A	0.119 (7)	0.123 (7)	0.100 (7)	−0.050 (7)	−0.026 (6)	−0.025 (5)
C6A	0.0470 (15)	0.0682 (19)	0.074 (3)	−0.0123 (11)	−0.0116 (13)	0.0018 (15)
C34B	0.063 (4)	0.077 (6)	0.086 (8)	−0.002 (4)	−0.007 (5)	0.023 (6)
C33B	0.083 (4)	0.090 (7)	0.071 (6)	0.012 (5)	−0.006 (4)	0.023 (4)
C32B	0.117 (11)	0.074 (7)	0.064 (7)	0.002 (7)	0.014 (7)	0.019 (6)
C31B	0.083 (8)	0.065 (2)	0.071 (3)	−0.002 (4)	0.017 (5)	0.012 (2)
C30B	0.061 (5)	0.061 (2)	0.057 (3)	0.001 (4)	0.013 (4)	0.002 (2)
C29B	0.058 (5)	0.061 (5)	0.056 (10)	0.003 (4)	0.005 (5)	0.009 (5)
C6B	0.0476 (18)	0.069 (2)	0.075 (3)	−0.0122 (14)	−0.0121 (16)	0.0012 (17)
O6B	0.153 (15)	0.131 (10)	0.104 (10)	−0.083 (12)	−0.013 (8)	−0.021 (8)
C12A	0.081 (7)	0.050 (4)	0.059 (6)	0.002 (4)	−0.005 (5)	0.006 (4)
C13A	0.100 (7)	0.056 (4)	0.068 (6)	0.000 (3)	−0.018 (4)	0.005 (4)
C14A	0.095 (7)	0.061 (5)	0.059 (3)	0.003 (4)	−0.015 (4)	0.002 (4)
C15A	0.098 (9)	0.065 (8)	0.061 (6)	−0.007 (6)	−0.020 (6)	0.006 (5)
C16A	0.080 (6)	0.055 (5)	0.045 (5)	0.003 (5)	−0.007 (5)	−0.003 (4)
C11A	0.065 (3)	0.0536 (18)	0.0453 (17)	0.0068 (19)	0.0034 (18)	−0.0023 (13)
C18A	0.091 (7)	0.072 (9)	0.115 (16)	−0.003 (6)	0.013 (8)	−0.042 (9)
C19A	0.103 (9)	0.059 (3)	0.102 (5)	−0.008 (5)	0.024 (7)	−0.028 (3)
C20A	0.088 (9)	0.054 (6)	0.081 (8)	0.007 (7)	0.001 (7)	−0.019 (5)
C21A	0.088 (6)	0.057 (7)	0.063 (9)	0.021 (5)	−0.007 (5)	−0.025 (5)
C22A	0.079 (2)	0.052 (3)	0.063 (6)	0.010 (2)	0.005 (3)	−0.019 (2)
C17A	0.080 (2)	0.0450 (18)	0.0477 (18)	−0.0005 (16)	0.0039 (16)	−0.0042 (14)
C23A	0.067 (8)	0.060 (8)	0.057 (8)	0.003 (6)	−0.021 (6)	−0.008 (6)
C24A	0.072 (5)	0.040 (7)	0.066 (4)	−0.010 (4)	0.017 (3)	0.010 (3)
C25A	0.108 (8)	0.072 (8)	0.071 (4)	0.000 (5)	0.032 (5)	0.013 (4)
C26A	0.138 (7)	0.096 (5)	0.053 (3)	0.007 (4)	0.013 (3)	−0.010 (3)
C27A	0.105 (6)	0.111 (8)	0.054 (3)	−0.001 (5)	−0.008 (3)	−0.022 (4)
C28A	0.078 (5)	0.101 (8)	0.054 (3)	−0.002 (5)	−0.007 (3)	−0.017 (4)
C23B	0.058 (9)	0.057 (9)	0.040 (9)	−0.007 (7)	0.026 (6)	0.003 (6)
C24B	0.065 (6)	0.031 (7)	0.059 (5)	−0.012 (4)	−0.005 (4)	0.004 (4)
C25B	0.076 (8)	0.060 (8)	0.071 (7)	−0.003 (5)	0.033 (6)	−0.001 (5)
C26B	0.138 (7)	0.096 (5)	0.053 (3)	0.007 (4)	0.013 (3)	−0.010 (3)
C27B	0.105 (6)	0.111 (8)	0.054 (3)	−0.001 (5)	−0.009 (3)	−0.022 (4)
C28B	0.075 (7)	0.081 (9)	0.054 (5)	−0.015 (6)	−0.015 (5)	0.007 (5)
C30A	0.061 (5)	0.061 (3)	0.057 (3)	0.002 (4)	0.013 (4)	0.002 (2)
C31A	0.083 (8)	0.065 (2)	0.071 (3)	−0.002 (4)	0.017 (5)	0.012 (2)
C32A	0.096 (9)	0.103 (9)	0.071 (8)	0.035 (8)	0.013 (6)	0.036 (7)
C33A	0.083 (4)	0.090 (7)	0.071 (6)	0.012 (5)	−0.006 (4)	0.023 (4)
C34A	0.059 (5)	0.089 (8)	0.058 (5)	0.010 (5)	−0.002 (4)	0.015 (6)
C29A	0.052 (5)	0.055 (5)	0.037 (7)	0.005 (4)	0.015 (4)	0.001 (4)
C5B	0.0470 (15)	0.0682 (19)	0.074 (3)	−0.0123 (11)	−0.0116 (13)	0.0018 (15)
O5B	0.0727 (14)	0.090 (2)	0.117 (2)	0.0196 (13)	0.0055 (15)	0.0113 (16)

### Geometric parameters (Å, º)

**Table:**

Os1—Os2	2.7494 (2)	C31B—H31B	0.9300
Os1—C10	2.087 (3)	C31B—C30B	1.3900
Os1—C7	2.104 (3)	C30B—H30B	0.9300
Os1—C1	1.946 (4)	C30B—C29B	1.3900
Os1—C2	1.891 (4)	C6B—O6B	1.136 (6)
Os1—C3	1.936 (4)	C12A—H12A	0.9300
Os2—C10	2.355 (3)	C12A—C13A	1.3900
Os2—C7	2.341 (3)	C12A—C11A	1.3900
Os2—C9	2.285 (3)	C13A—H13A	0.9300
Os2—C8	2.279 (3)	C13A—C14A	1.3900
Os2—C4	1.911 (4)	C14A—H14A	0.9300
Os2—C5A	1.920 (5)	C14A—C15A	1.3900
Os2—C6A	1.922 (4)	C15A—H15A	0.9300
Os2—C6B	1.922 (4)	C15A—C16A	1.3900
Os2—C5B	1.920 (5)	C16A—H16A	0.9300
O2—C2	1.137 (4)	C16A—C11A	1.3900
O1—C1	1.134 (4)	C18A—H18A	0.9300
C10—C9	1.420 (4)	C18A—C19A	1.3900
C10—C11B	1.526 (11)	C18A—C17A	1.3900
C10—C11A	1.482 (10)	C19A—H19A	0.9300
O3—C3	1.135 (5)	C19A—C20A	1.3900
C7—C8	1.419 (5)	C20A—H20A	0.9300
C7—C29B	1.563 (10)	C20A—C21A	1.3900
C7—C29A	1.462 (10)	C21A—H21A	0.9300
O4—C4	1.131 (5)	C21A—C22A	1.3900
C9—C8	1.461 (5)	C22A—H22A	0.9300
C9—C17B	1.499 (4)	C22A—C17A	1.3900
C9—C17A	1.499 (4)	C23A—C24A	1.3900
C8—C23A	1.504 (4)	C23A—C28A	1.3900
C8—C23B	1.504 (4)	C24A—H24A	0.9300
O5A—C5A	1.190 (8)	C24A—C25A	1.3900
C22B—H22B	0.9300	C25A—H25A	0.9300
C22B—C21B	1.3900	C25A—C26A	1.3900
C22B—C17B	1.3900	C26A—H26A	0.9300
C21B—H21B	0.9300	C26A—C27A	1.3900
C21B—C20B	1.3900	C27A—H27A	0.9300
C20B—H20B	0.9300	C27A—C28A	1.3900
C20B—C19B	1.3900	C28A—H28A	0.9300
C19B—H19B	0.9300	C23B—C24B	1.3900
C19B—C18B	1.3900	C23B—C28B	1.3900
C18B—H18B	0.9300	C24B—H24B	0.9300
C18B—C17B	1.3900	C24B—C25B	1.3900
C16B—H16B	0.9300	C25B—H25B	0.9300
C16B—C15B	1.3900	C25B—C26B	1.3900
C16B—C11B	1.3900	C26B—H26B	0.9300
C15B—H15B	0.9300	C26B—C27B	1.3900
C15B—C14B	1.3900	C27B—H27B	0.9300
C14B—H14B	0.9300	C27B—C28B	1.3900
C14B—C13B	1.3900	C28B—H28B	0.9300
C13B—H13B	0.9300	C30A—H30A	0.9300
C13B—C12B	1.3900	C30A—C31A	1.3900
C12B—H12B	0.9300	C30A—C29A	1.3900
C12B—C11B	1.3900	C31A—H31A	0.9300
O6A—C6A	1.136 (6)	C31A—C32A	1.3900
C34B—H34B	0.9300	C32A—H32A	0.9300
C34B—C33B	1.3900	C32A—C33A	1.3900
C34B—C29B	1.3900	C33A—H33A	0.9300
C33B—H33B	0.9300	C33A—C34A	1.3900
C33B—C32B	1.3900	C34A—H34A	0.9300
C32B—H32B	0.9300	C34A—C29A	1.3900
C32B—C31B	1.3900	C5B—O5B	1.027 (11)
			
C10—Os1—Os2	56.30 (8)	C13B—C14B—H14B	120.0
C10—Os1—C7	77.63 (12)	C14B—C13B—H13B	120.0
C7—Os1—Os2	55.80 (8)	C14B—C13B—C12B	120.0
C1—Os1—Os2	107.09 (11)	C12B—C13B—H13B	120.0
C1—Os1—C10	92.72 (14)	C13B—C12B—H12B	120.0
C1—Os1—C7	162.88 (14)	C11B—C12B—C13B	120.0
C2—Os1—Os2	149.94 (10)	C11B—C12B—H12B	120.0
C2—Os1—C10	103.52 (13)	C16B—C11B—C10	113.7 (10)
C2—Os1—C7	100.99 (13)	C12B—C11B—C10	126.2 (10)
C2—Os1—C1	94.97 (15)	C12B—C11B—C16B	120.0
C2—Os1—C3	94.53 (16)	O5A—C5A—Os2	177.8 (8)
C3—Os1—Os2	104.74 (12)	O6A—C6A—Os2	175.1 (11)
C3—Os1—C10	160.96 (15)	C33B—C34B—H34B	120.0
C3—Os1—C7	93.18 (15)	C33B—C34B—C29B	120.0
C3—Os1—C1	91.69 (16)	C29B—C34B—H34B	120.0
C10—Os2—Os1	47.50 (8)	C34B—C33B—H33B	120.0
C7—Os2—Os1	48.00 (8)	C32B—C33B—C34B	120.0
C7—Os2—C10	68.03 (11)	C32B—C33B—H33B	120.0
C9—Os2—Os1	72.85 (8)	C33B—C32B—H32B	120.0
C9—Os2—C10	35.61 (11)	C33B—C32B—C31B	120.0
C9—Os2—C7	62.97 (12)	C31B—C32B—H32B	120.0
C8—Os2—Os1	73.20 (8)	C32B—C31B—H31B	120.0
C8—Os2—C10	63.01 (11)	C30B—C31B—C32B	120.0
C8—Os2—C7	35.75 (11)	C30B—C31B—H31B	120.0
C8—Os2—C9	37.35 (12)	C31B—C30B—H30B	120.0
C4—Os2—Os1	95.33 (13)	C31B—C30B—C29B	120.0
C4—Os2—C10	89.41 (15)	C29B—C30B—H30B	120.0
C4—Os2—C7	143.26 (15)	C34B—C29B—C7	125.3 (8)
C4—Os2—C9	114.18 (15)	C30B—C29B—C7	114.7 (8)
C4—Os2—C8	151.01 (15)	C30B—C29B—C34B	120.0
C4—Os2—C5A	89.9 (3)	O6B—C6B—Os2	167.0 (17)
C4—Os2—C6A	95.4 (3)	C13A—C12A—H12A	120.0
C4—Os2—C6B	87.7 (4)	C13A—C12A—C11A	120.0
C4—Os2—C5B	93.7 (5)	C11A—C12A—H12A	120.0
C5A—Os2—Os1	87.3 (3)	C12A—C13A—H13A	120.0
C5A—Os2—C10	134.5 (3)	C14A—C13A—C12A	120.0
C5A—Os2—C7	86.6 (2)	C14A—C13A—H13A	120.0
C5A—Os2—C9	149.6 (2)	C13A—C14A—H14A	120.0
C5A—Os2—C8	115.4 (3)	C13A—C14A—C15A	120.0
C5A—Os2—C6A	91.5 (4)	C15A—C14A—H14A	120.0
C6A—Os2—Os1	169.2 (3)	C14A—C15A—H15A	120.0
C6A—Os2—C10	133.9 (3)	C16A—C15A—C14A	120.0
C6A—Os2—C7	121.3 (3)	C16A—C15A—H15A	120.0
C6A—Os2—C9	103.7 (3)	C15A—C16A—H16A	120.0
C6A—Os2—C8	97.8 (2)	C11A—C16A—C15A	120.0
C6B—Os2—Os1	165.6 (4)	C11A—C16A—H16A	120.0
C6B—Os2—C10	118.6 (4)	C12A—C11A—C10	119.4 (8)
C6B—Os2—C7	128.2 (3)	C16A—C11A—C10	120.3 (8)
C6B—Os2—C9	93.0 (4)	C16A—C11A—C12A	120.0
C6B—Os2—C8	97.5 (3)	C19A—C18A—H18A	120.0
C5B—Os2—Os1	101.0 (4)	C19A—C18A—C17A	120.0
C5B—Os2—C10	148.5 (4)	C17A—C18A—H18A	120.0
C5B—Os2—C7	92.0 (4)	C18A—C19A—H19A	120.0
C5B—Os2—C9	151.7 (4)	C18A—C19A—C20A	120.0
C5B—Os2—C8	114.4 (4)	C20A—C19A—H19A	120.0
C5B—Os2—C6B	92.9 (6)	C19A—C20A—H20A	120.0
Os1—C10—Os2	76.20 (11)	C19A—C20A—C21A	120.0
C9—C10—Os1	117.1 (2)	C21A—C20A—H20A	120.0
C9—C10—Os2	69.49 (18)	C20A—C21A—H21A	120.0
C9—C10—C11B	117.1 (10)	C22A—C21A—C20A	120.0
C9—C10—C11A	117.5 (9)	C22A—C21A—H21A	120.0
C11B—C10—Os1	125.5 (10)	C21A—C22A—H22A	120.0
C11B—C10—Os2	129.9 (7)	C21A—C22A—C17A	120.0
C11A—C10—Os1	125.3 (9)	C17A—C22A—H22A	120.0
C11A—C10—Os2	125.8 (7)	C18A—C17A—C9	118.8 (10)
Os1—C7—Os2	76.20 (10)	C22A—C17A—C9	121.1 (10)
C8—C7—Os1	116.8 (2)	C22A—C17A—C18A	120.0
C8—C7—Os2	69.73 (17)	C24A—C23A—C8	117.8 (5)
C8—C7—C29B	120.6 (6)	C24A—C23A—C28A	120.0
C8—C7—C29A	118.3 (5)	C28A—C23A—C8	122.2 (5)
C29B—C7—Os1	122.1 (6)	C23A—C24A—H24A	120.0
C29B—C7—Os2	131.1 (5)	C23A—C24A—C25A	120.0
C29A—C7—Os1	124.8 (5)	C25A—C24A—H24A	120.0
C29A—C7—Os2	127.3 (5)	C24A—C25A—H25A	120.0
C10—C9—Os2	74.90 (19)	C26A—C25A—C24A	120.0
C10—C9—C8	114.4 (3)	C26A—C25A—H25A	120.0
C10—C9—C17B	123.5 (10)	C25A—C26A—H26A	120.0
C10—C9—C17A	123.9 (10)	C25A—C26A—C27A	120.0
C8—C9—Os2	71.11 (18)	C27A—C26A—H26A	120.0
C8—C9—C17B	120.9 (10)	C26A—C27A—H27A	120.0
C8—C9—C17A	120.8 (10)	C28A—C27A—C26A	120.0
C17B—C9—Os2	132.5 (7)	C28A—C27A—H27A	120.0
C17A—C9—Os2	131.7 (7)	C23A—C28A—H28A	120.0
C7—C8—Os2	74.52 (18)	C27A—C28A—C23A	120.0
C7—C8—C9	114.1 (3)	C27A—C28A—H28A	120.0
C7—C8—C23A	127.4 (4)	C24B—C23B—C8	115.7 (7)
C7—C8—C23B	122.0 (4)	C24B—C23B—C28B	120.0
C9—C8—Os2	71.54 (18)	C28B—C23B—C8	124.3 (7)
C9—C8—C23A	117.7 (4)	C23B—C24B—H24B	120.0
C9—C8—C23B	123.5 (5)	C25B—C24B—C23B	120.0
C23A—C8—Os2	131.0 (4)	C25B—C24B—H24B	120.0
C23B—C8—Os2	128.6 (4)	C24B—C25B—H25B	120.0
O1—C1—Os1	179.8 (4)	C24B—C25B—C26B	120.0
O2—C2—Os1	178.2 (3)	C26B—C25B—H25B	120.0
O3—C3—Os1	178.7 (4)	C25B—C26B—H26B	120.0
O4—C4—Os2	178.2 (4)	C27B—C26B—C25B	120.0
C21B—C22B—H22B	120.0	C27B—C26B—H26B	120.0
C21B—C22B—C17B	120.0	C26B—C27B—H27B	120.0
C17B—C22B—H22B	120.0	C26B—C27B—C28B	120.0
C22B—C21B—H21B	120.0	C28B—C27B—H27B	120.0
C22B—C21B—C20B	120.0	C23B—C28B—H28B	120.0
C20B—C21B—H21B	120.0	C27B—C28B—C23B	120.0
C21B—C20B—H20B	120.0	C27B—C28B—H28B	120.0
C19B—C20B—C21B	120.0	C31A—C30A—H30A	120.0
C19B—C20B—H20B	120.0	C31A—C30A—C29A	120.0
C20B—C19B—H19B	120.0	C29A—C30A—H30A	120.0
C20B—C19B—C18B	120.0	C30A—C31A—H31A	120.0
C18B—C19B—H19B	120.0	C30A—C31A—C32A	120.0
C19B—C18B—H18B	120.0	C32A—C31A—H31A	120.0
C19B—C18B—C17B	120.0	C31A—C32A—H32A	120.0
C17B—C18B—H18B	120.0	C33A—C32A—C31A	120.0
C22B—C17B—C9	111.9 (10)	C33A—C32A—H32A	120.0
C18B—C17B—C9	127.9 (10)	C32A—C33A—H33A	120.0
C18B—C17B—C22B	120.0	C32A—C33A—C34A	120.0
C15B—C16B—H16B	120.0	C34A—C33A—H33A	120.0
C15B—C16B—C11B	120.0	C33A—C34A—H34A	120.0
C11B—C16B—H16B	120.0	C29A—C34A—C33A	120.0
C16B—C15B—H15B	120.0	C29A—C34A—H34A	120.0
C16B—C15B—C14B	120.0	C30A—C29A—C7	118.3 (8)
C14B—C15B—H15B	120.0	C34A—C29A—C7	121.7 (8)
C15B—C14B—H14B	120.0	C34A—C29A—C30A	120.0
C13B—C14B—C15B	120.0	O5B—C5B—Os2	172.8 (12)

### Hydrogen-bond geometry (Å, º)

**Table:**

*D*—H···*A*	*D*—H	H···*A*	*D*···*A*	*D*—H···*A*
C19*A*—H19*A*···O2^i^	0.93	2.60	3.363 (12)	139

Symmetry code: (i) −*x*+1, *y*+1/2, −*z*+1/2.
